# Does perinatal period pelvic floor muscle exercises affect sexuality and pelvic muscle strength? A systematic review and meta-analysis of randomized controlled trials

**DOI:** 10.1590/1806-9282.20220133

**Published:** 2022-08-19

**Authors:** Aysu Yıldız Karaahmet, Nuran Gençturk, Nur E lcin Boyacıoğlu

**Affiliations:** 1 Haliç University, School of Health Sciences – Beyoğlu, Turkey.; 2 Istanbul University, Faculty of Health Science, Department of Midwifery – Beyoğlu, Turkey.; 3 Istanbul University, Faculty of Health Science, Department of Midwifery – Büyükçekmece, Turkey.

**Keywords:** Pregnancy, Postpartum, Sexuality, Exercise, Meta-analysis

## Abstract

**OBJECTIVE:**

The aim of this study was to systematically review the effect of pelvic floor exercises on female sexual function and pelvic floor strength in the prenatal and postnatal periods and to conduct a meta-analysis of available evidence.

**METHODS:**

Published archives, including PubMed, Cochrane Library, Web of Science, and ULAKBİM databases, were scanned using keywords based on MeSH. Only randomized controlled trials were included. The data were analyzed using the Review Manager computer program (version 5.3).

**RESULTS:**

Pooled standardized differences in means of sexual function in both pelvic floor exercise and control group were 6.33 (95%CI 5.27–7.40, p<0.00001) during pregnancy. The pooled standardized differences in means in sexual function after postpartum intervention was 1.19 (95%CI 0.08––2.30, p=0.04).

**CONCLUSION:**

Evidence has shown a little effect on the pelvic floor muscle training on sexual function in pregnancy and postpartum period in primipara women, and it is a safe strategy that can improve postpartum sexual function.

## INTRODUCTION

Sexuality is a natural and important part of human life^1,2^. Sexual dysfunction is defined as a disorder affecting sexual desire that can result in interpersonal difficulties, pronounced distress, and psychophysiological changes^3,4^.

The etiology of female sexual dysfunction has a multifactorial structure^5–7^. Especially during pregnancy and after delivery, deterioration of pelvic muscle strength (PMS) is an important risk factor^3,7,8^. The literature has shown that pelvic floor muscle exercise (PFME) can improve sexual desire and orgasm capacity in the general population and in women with weak orgasm problems caused by poor pelvic muscle tone^8,9^. The literature on the effects of PFME on female sexual function (SF) has limited edition reviewed, especially for its efficacy during pregnancy and postpartum, and only two studies were meta-analyzed^6,7^. Therefore, the aim of this study was to systematically review the effect of PME on female SF and PMS in the prenatal and postnatal periods and to conduct a meta-analysis of available evidence.

## METHODS

Systematic examination and meta-analysis of the studies evaluating the effect of PMFE on female SFs and pelvic floor strength in the prenatal and postnatal periods were performed. In the preparation of systematic review and meta-analysis, the criteria in the PRISMA and *Cochrane Experiments Systematic Reviews Handbook* were used.

### Search strategy

A comprehensive, systematic search of PubMed, Web of Science, the Cochrane Library, and ULAKBİM databases was completed from the earliest date available until February 2020. The Web of Science Core Collection was searched using the following keywords: “pelvic floor exercise” OR “pelvic muscle strength” OR “sexual functions” AND “pregnancy” OR “postpartum.” The search strategy was changed according to the characteristics of each database.

### Inclusion and Exclusion Criteria

The inclusion and exclusion criteria used were as follows:

(1) only randomized controlled trials (RCTs) were included in the study;
(2) in the intervention group, pelvic floor exercises to improve the pelvic floor were included if Kegel, Pilates, or yoga were used;
(3) studies that included effects of PFME on at least one SF variable including prenatal or postpartum desire, arousal, orgasm, pain, lubrication, and satisfaction; and
(4) studies published only in English and Turkish languages were included.

### Study selection and data extraction

After the duplicate articles retrieved from the different databases were removed, two independent researchers (A.Y.K. and N.E.B.) screened titles and abstracts to identify which studies met the inclusion and exclusion criteria. If there was a contradiction between the researchers, the third researcher (N.G.) was assisted to reach an agreement. Data were obtained using standard data extraction forms including study characteristics (i.e., design, population, experiments, and result), PICOS (participant, experiment, comparison, outcomes, and study design) approach, age, gender, and follow-up time (Table 1).

**Table 1.** Study characteristics.

Author (reference)\Publication date\Country	Study type	population	Training protocol	Comparisons	Drop out	Outcome measures	Results
Wang et al.^17^	RCT	108 primiparous woman 1.Audio guidance group (n=54) 2-Control Group (n=54)	Participants in the intervention group received audio guidance training. – The pelvic floor muscle training practice guide is as follows: participants were directed to lie in the supine position bending the hips and knees and to relax the abdomen and hip muscles while selectively contracting and breathing the urethra, vagina and anus muscles. Meanwhile, the researchers were asked to evaluate on the abdomen, and on the other hand, by placing the participants on their hips and/and middle fingers in their vagina with postpartum sterile gloves to determine if the participants could properly contract and contract these muscles. – The general principles of training were: training at least two times per day and 15 minutes per time, or 150 contractions per day; keep training for at least 3 months. Researchers called the participants of the two groups once a month to answer their questions related to training or stress incontinence and encourage them to keep on training for at least 3 months.	The control group eceiving conventional home-based training through random looting.	– Interventions group (n=6) – Control group (n=4)	FSFI	Participants were not significant in 6 groups postpartum sexual function in both groups during the study (p=0.007)
Pourkhiz et al.^10^ İran	RCT	84 primiparous woman 1-PFM training group (n=41) 2-usual care group (n=41)	PFM: at least twice a day, 8–12 contractions at each time, holding for 6–8 s. Start from 36 to 37 weeks and after giving birth as soon as one can FSFI SQOL-F: the mean total sexual function and sexual quality of life score was greater in the PFM training group; (p < 0.001) One in each group	Control: routine care	– PFM (n=1) – Control (n=1) One in each group	FSFI SQOL-F	the mean total sexual function and sexual quality of life score was greater in the PFM training group; (p<0.001)
Tennfjord et al.^15^ Norway	RCT	175 primiparous woman 1-PFMT training group (n=87) 2-control group (n=88)	- The training group attended a weekly PFMT class for 4 months, starting 6 weeks postpartum. Also they did daily three sets of 8–12 PFM contractions at home. At 6 weeks (baseline) and 6 months postpartum women answered an electronic questionnaire.	-Control: routine care	– Interventions group (n=3) – Control groups (n=10)	ICIQ-FLUTSsex	No difference was seen between case and control groups in symptoms related to sexual dysfunction during 6 months of postpartum
Oakley et al.^11^, Ohio	RCT	50 primiparous woman 1-PFPT group (n=27), 2-control group (n=23)	- The intervention group did PFPT with biofeedback and received Behavioral therapy. The intervention arm completed four 60-min PFPT sessions which beginning at week 6 after delivery. Both groups completed questionnaires as well at baseline (week 2) and weeks 12 postpartum.	Control: routine care	– Interventions group (n=2) – Control group (n=2)	FSFI, FIQOL, SF-12, UDI-6, IIQ-7, FISI	Both groups significantly improved in physical health (p<0.000) and sexual function (p<0.000) after 12 weeks of postpartum, but there was no difference between two groups.
Golmakani et al.^16^, Meşhed, İran	RCT	79 primiparous woman 1-PFMT training group (n=40) 2-control group (n=39)	After 8 week of delivery women were trained to Contract their pelvic floor muscles for 5–10 s and relax for 5–10 s and repeating this exercise for 20 times (for 5 min). After 2 min of rest, they again had to perform this exercise for 3 times of 5 min. so that a total of 20 min of exercise is performed at each time. Twice daily, each time 15–20 times depending on their ability. 4 and 8 weeks after the beginning of the study compared in both group	Control: routine care	–Interventions group (n=12) – Control group (n=13)	Brink scale, Bailes sexual self-efficacy	Comparison of the two groups presented a significant difference in sexual self-efficacy after performing these exercises (p=0.001).
Haruna et al.^14^, Tokyo	RCT	95 primiparous woman 1-Intervention group; (n=48), 2-control group; (n=47)	Exercise classes were held 4 times weekly for 4 weeks, 90 min each, at three months postpartum. The exercise class included: aerobic exercise (50–60 min) where the participant sits and bounces on an exercise ball. The outcome measures were assessed at two months postpartum (baseline) and at four months postpartum (outcome).	Control: routine care	– Interventions group (n=2) – Control group (n=4)	SF-36v2	The postpartum exercise class improved health-related QOL in the training group compared to the control group, although there were no significant differences in the Physical and Mental component of quality of life between the groups.
Çıtak et al.^12^, Turkey	RCT	75 primiparous woman 1-PFM training group (n=37) 2-control group (n=38)	- In the 4th postpartum month women were trained to do PFM contraction. 2–3 s contraction and relaxation, ten times a day in the first 15 days. Thereafter, the duration of contraction and relaxation was changed to five seconds. Then increase the durations to 10seconds and the number of workouts to 15 sessions/day up to the end of the study. The results of both groups, obtained in the 4th and 7th postpartum months, were compared	Control: routine care	–Interventions group (n=21) – Control group (n=22)	FSFI	All domains except satisfaction were significantly higher in the training group compared with the controls. sexual arousal, lubrication, orgasm, and satisfaction scores were improved in the 7th month in the training group;(P < 0.001)
Reilly et al.^13^, UK	RCT	230 primiparous woman 1-PFE training group (n=120) 2-control group (n=110)	The exercises comprised three repetitions of eight contractions each held for six seconds, with two minutes rest between repetitions. These were repeated twice daily. At 34 weeks of gestation the number of contractions per repetition was increased to 12.	Control: routine care	– Interventions group (n=52) –Control group (n=47)	King’s Health Questionnaire, SF-36	At 3 months, there was no difference between the intervention groups on any of the eight scales of the Kings Health Questionnaire. higher score for the general health measure in the Short Form-36 in those in the exercise group compared with the control group

### Risk of bias assessment

The quality of the selected articles was evaluated by two researchers (A.Y.K. and N.E.B.) with the Quality Assessment Tool (EPHPP) checklist. The evaluation of the risk of bias of all selected articles was done by two authors (A.Y.K. and N.B.E.) independently using modified Cochrane tools for assessing risk of bias, following the criteria outlined in the *Cochrane Handbook for Systematic Reviews of Interventions*. The other author (N.G.) checked the results. Risk of bias was classified into seven domains. The bias risk for each area was classified as “low risk,” “high risk,” or “uncertain risk,” according to the decision criteria in the “Risk of bias” assessment tool.

### Quantitative data synthesis and analysis

Outcomes data including SF and PMS of the participants who had used PMEs were collected for analysis. Meta-analysis of study outcomes was performed using RevMan version 5.3.

For analysis of continuous data, mean differences (MD) or standardized mean differences (SMD) with 95% confidence intervals (CI) were used. SMD was used when the studies assess the same outcome but measure it in a variety of ways (e.g., all studies measure function, but they use different psychometric scales such as Female Sexual Functıon Index [FSFI] and Golombok-Rust Sexual Satisfaction Scale [GRISS]). Statistical heterogeneity was determined by I^2^. A value of 0% indicates no observed heterogeneity, and larger values indicate increased heterogeneity. Coherence between researchers for independent article selection and bias scores was evaluated using the Cohen’s kappa statistic. Only 62.5% (n=5) of the studies were graded 1 according to the EPHPP tool. Coherence between the observers was excellent both in the selection of articles and in the scoring of selected articles in terms of bias (Cohen’s kappa was 0.95 for article selection and 0.97 for bias scoring; p=0.000).

## RESULTS

### Literature search

**Figure 1.**
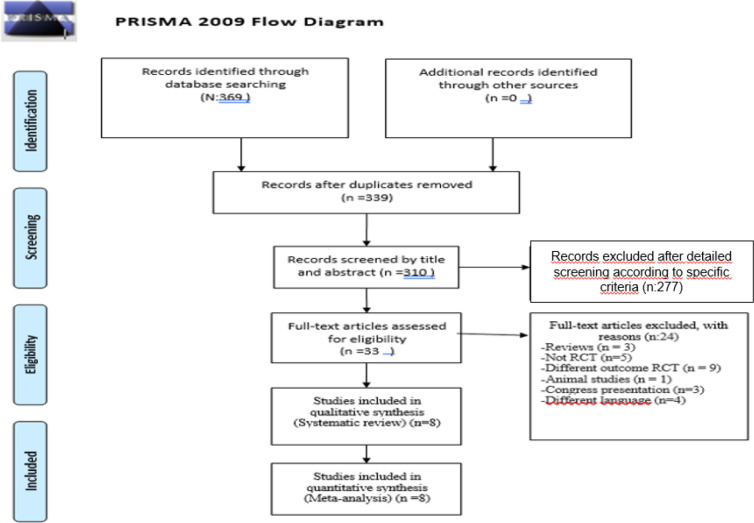
PRISMA flow diagram of selection of study process.

The PRISMA flowchart for searching and selecting literature is summarized in Figure 1.

The electronic database search and hand search yielded 369 potentially relevant studies. After removing duplicates, we screened 339 articles based on title or abstract. The remaining 33 full texts were assessed for eligibility. For the full-text screening, a third reviewer was needed to resolve disagreements, all regarding the blinding of the studies. Eight trials met all eligibility criteria and were included in qualitative synthesis (Figure 1).

### Study characteristics

Eight trials (896 participants in total) were included in these reviews and meta-analysis^10–17^. The features of the studies are summarized in Table 1. All other studies started in the postpartum period except for the two studies (started during pregnancy)^10–13^. The duration of the experiments varies between 4 and 20 weeks. In most of the articles, women in the control group received routine postpartum care. However, in one study, the control group received conventional home-based training^17^. Women in the intervention group received the following treatments: those in Wang et al^17^ received audio guidance training; those in Oakley et al^11^ received biofeedback with pelvic floor exercise; those in Golmakoni et al.^16^ received Kegel exercises; those in Haruna et al^14^ received aerobic exercise; and those in the remaining four studies received PFME only. The entire patient population included primiparous women. In most studies, SF states were evaluated as the primary outcome. Two of the studies^10–13^ evaluated SF in both pregnancy and postpartum period, while all other studies evaluated SF in the postpartum period. All studies included in the review have been reported on SF in the postpartum period.

### Outcome Measures

**Figure 2.**
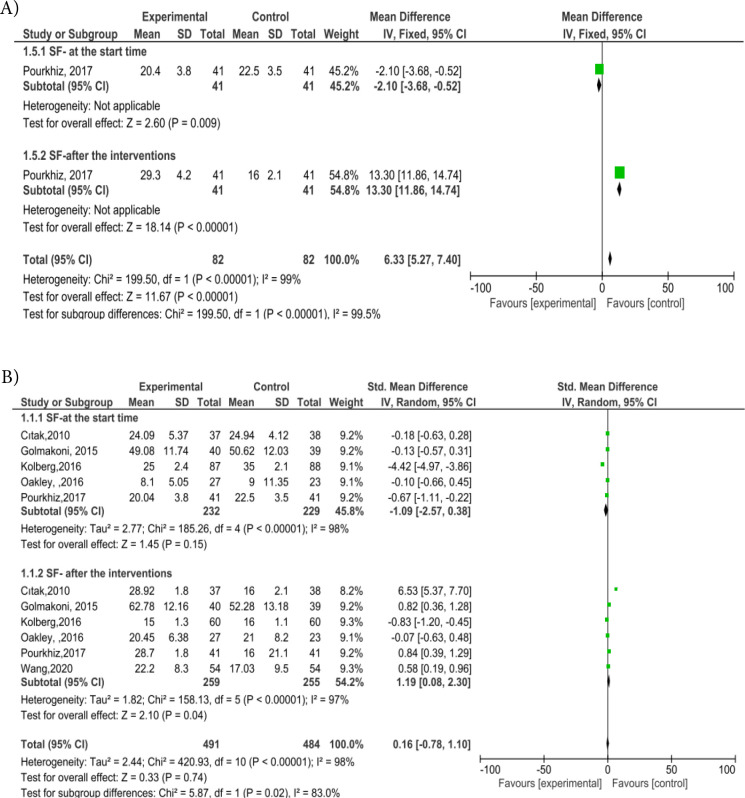
Effect of prenatal and postnatal pelvic floor muscle exercise on sexual function forest plot. A: Effect of prenatal pelvic floor muscle exercise on sexual function forest plot. B: Effect of postnatal pelvic floor muscle exercise on sexual function forest plot.

The forest graphic in Figure 2 shows us the meta-analysis of the effect of PMFE on SF. Four studies used the FSFI questionnaire^10–12,17^ to evaluate SF.

### Effect of exercises on SF

Six articles^10–12,15–17^ reporting on SF were included in the meta-analysis. In the prenatal period, only one study reported sexual results. Figure 2 shows the effects of pelvic floor exercises on SF during pregnancy. The study^10^, which included 82 participants in total (41 receiving PFME), examined the effects of PFME on SF. Pooled SMDs of SF in both PME and control groups were 6.33 (95%CI 5.27–7.40, p<0.00001).

**Figure 3.**
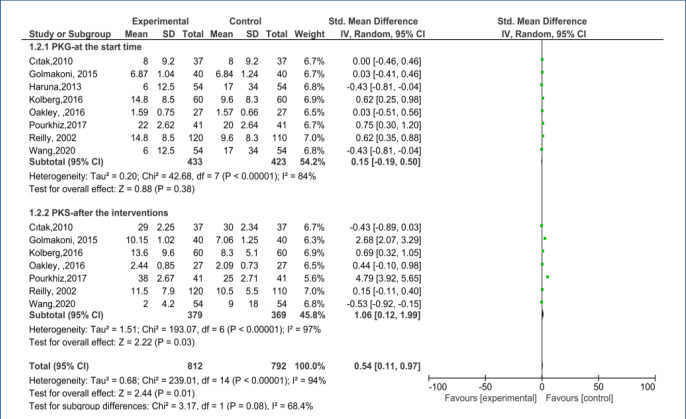
Effect of pelvic floor muscle exercise on pelvic floor strength forest plot.

When we evaluated PFME sexual status in the postpartum period, based on the random-effects model, SMDs of SF in both PME and control groups were 1.19 (95%CI 0.08–2.30, p=0.04). A meta-analysis of these studies revealed that PME can improve SF in the postpartum period. The included studies had high heterogeneity (I2=83.0%; p=0.02). The forest plot is shown in Figure 3.

### Pelvic floor muscle strength

In the three studies,^10–12^ the Oxford grading system, an accepted international method for determining the strength of the pelvic floor muscles (PFMs), was used. Other studies have evaluated PMS with different assessment tools. Eight articles have reported PMS-related results and are included in the meta-analysis^10–12,15–17^. The PMS SMDs in both groups were 1.06 (95%CI 0.12–1.99, p=0.03). Meta-analysis of these studies showed a significant relationship between PFME and PMS. The included studies had high heterogeneity (I^2^=94.0%, p<0.00001). The forest plot of the meta-analysis is shown in Figure 3.

### Risk of bias assessment

**Figure 4.**
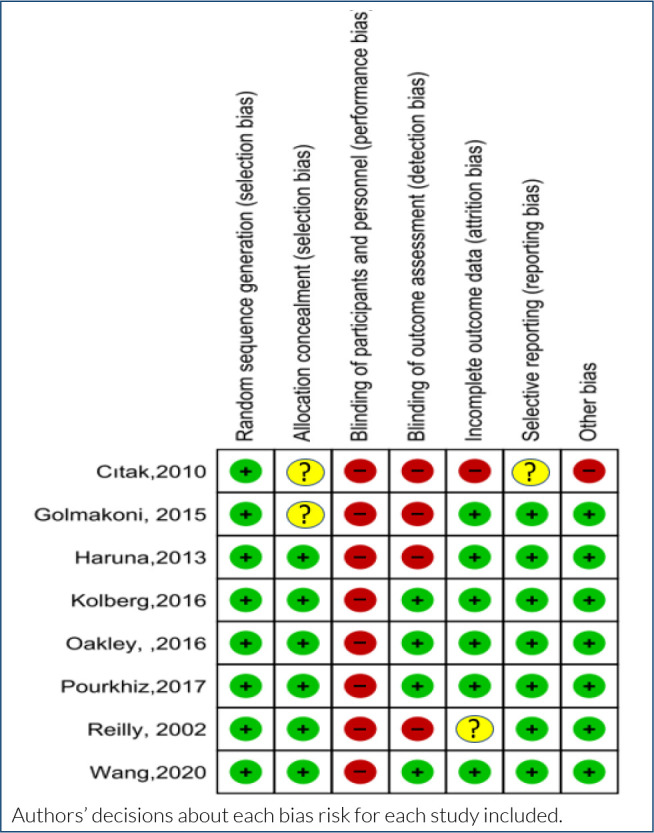
Risk of bias summary.

All studies have identified a sufficient method for random allocation of participants to exercise groups^10–17^. Six studies reported adequate allocation confidentiality using sequentially numbered and sealed opaque envelopes^10–12,14,15,17^ and evaluated them at low risk of bias. In all studies included in the meta-analysis, it was not possible for the participants and researchers participating in the experiment to be blind to the study.^10–12,14,15,17^ Four studies are at low risk for blinding outcome evaluation.^10,11,15,17^ Other studies have also been evaluated without blinding the outcome assessment and as at a high risk of bias.^12–14,16^ In these six studies, the drop-outs were balanced between the intervention and control groups, or there were too few drop-outs to affect the study.^10,11,14–17^ Apart from the study by Cıtak et al^12^ (uncertainty bias risk), in all other methods of work, they were evaluated at the risk of reporting low bias, as they discussed important reported results, including negative results and match those reported in their records or protocols^10,11,15^. In particular, we expected a conflict of interest statement and a source of funding. None of the included studies reported other bias risk (Figure 4).

## DISCUSSION

The purpose of this meta-analysis was to evaluate the effectiveness of PFMEs on SF in women during pregnancy and the postpartum period. Across the included studies, we examined whether there is evidence that PFMEs improve SF and PMS during pregnancy or in the postpartum period.

Although there was a significant increase in SF status as a result of using PFMEs, which we considered as the primary outcome in the examination, the evidence was generally of low quality. Therefore, we needed higher quality RCTs in this area to provide a more definitive answer. In addition to the study by Pourkhiz et al^10^, eight studies on PFMEs do not provide sufficient data to evaluate the effect on SF during pregnancy, but according to the analysis of studies in postpartum period, the use of PFMEs resulted in a statistically significant increase in SF^11,12,15^. Although most studies show an improvement in SF, the results should be interpreted with caution due to the methodological limitations of some studies. Çıtak et al^12^ had a high rate of attrition. However, high heterogeneity between studies is remarkable. Hadizadeh-Talasaz et al.^18^ in their meta-analysis found that women who performed postpartum PFMEs showed a slight improvement in SF problems. Wu et al.^19^ reported a decrease in unsatisfactory SF in their meta-analysis.

According to some studies, in addition to sexual dysfunction during pregnancy and the prevalence of PMS, incontinence and quality of life are increasing^19,20^. The effect of PFME on PMS, incontinence, and quality of life during pregnancy is an important research area.^20,21^ Our meta-analysis showed that after PFMEs, there was a significant improvement in PFM strength and quality of life, while a single study for incontinence showed no significant relationship with PFMEs. In addition, we determined that the quality of the evidence was from low to average, respectively. Studies on the pelvic floor during pregnancy revealed a clear link between pelvic floor disorder and low PMS^20^. Physical and hormonal changes caused by pregnancy are factors that can reduce PMS by affecting the pelvic floor. Although all studies have reported improvement in PMS, further studies are needed due to the low number of studies in this field and the low quality of evidence^21^.

## CONCLUSION

Evidence has shown a less effect on the PFM training on SF in pregnancy and postpartum period in primipara women, and it is a safe strategy that can improve postpartum SF.

PFME during pregnancy can prevent pelvic structure disorders and negative effects of sexuality in the later stages of pregnancy. However, study populations and quality of evidence are low. Although most studies and meta-analysis results show positive results, higher quality RCTs are needed in this area.
